# Supplementary material for: Proteotranscriptomic Profiling of the Toxic Mucus of Kulikovia alborostrata (Pilidiophora, Nemertea)
Source: Toxins (Basel). 2024 Dec 26;17(1):5. doi: 10.3390/toxins17010005 (PMC11768458; doi:10.3390/toxins17010005)
Supplement: Supplementary file 1 [file toxins-17-00005-s001.zip › Supplementary File S4.zip!/ORF 076246 LINALB/5646dc0998f06131/dom_summary.html]

Phyre 2 Results for putative\_toxin\_cistein\_rich\_

|  |  |
| --- | --- |
| Email | vas9i-kz@mail.ru |
| Description | putative\_toxin\_cistein\_rich\_ |
| Date | Wed Apr 5 07:02:50 BST 2023 || Unique Job ID | 5646dc0998f06131 |

|  |  |
| --- | --- |
| Domain analysis |  |

| Rank | Aligned region |
| --- | --- |

|  |  |
| --- | --- |
| 1 | --- \| c6enaA\_ \| --- |
| 2 | --- \| d1h5oa\_ \| --- |
| 3 | --- \| c1z99A\_ \| --- |
| 4 | --- \| d1vtxa\_ \| --- |
